# Editorial: Mendelian randomization and cardiovascular remodeling

**DOI:** 10.3389/fphar.2025.1593325

**Published:** 2025-03-25

**Authors:** Jianxin Qiang, Jia Yang, Yanwu Liu, Ji Zhang, Hao Sun, Lijuan Li, Duo Wang, Yanping Liu, Panpan Hao

**Affiliations:** ^1^ Department of Cardiology, Wuzhong People’s Hospital Affiliated to Ningxia Medical University, Wuzhong, Ningxia, China; ^2^ 120 First-aid Call Center, Wuzhong People’s Hospital Affiliated to Ningxia Medical University, Wuzhong, Ningxia, China; ^3^ Department of Neurology, Wuzhong People’s Hospital Affiliated to Ningxia Medical University, Wuzhong, Ningxia, China; ^4^ Department of Teaching and Research, Wuzhong People’s Hospital Affiliated to Ningxia Medical University, Wuzhong, Ningxia, China; ^5^ Department of Radiology, Qilu Hospital of Shandong University, Jinan, Shandong, China; ^6^ Department of Cardiology, Qilu Hospital of Shandong University, Jinan, Shandong, China

**Keywords:** mendelian randomization, cardiovascular remodeling, heart failure, diabetes, atheroclerosis

## 1 Introduction

Cardiovascular remodeling—a dynamic process of structural and functional adaptation in the heart and vasculature—is a hallmark of diseases ranging from heart failure to atherosclerosis (Heusch et al., 2014). Despite advances in treatment, its multifactorial etiology, driven by genetic predisposition, metabolic dysregulation, and environmental influences, remains incompletely understood. Mendelian randomization (MR), a method leveraging genetic variants as instrumental variables, has emerged as a powerful tool to disentangle causal relationships in observational data, offering unparalleled insights into disease mechanisms and therapeutic opportunities (Larsson et al., 2023). This Research Topic, *Mendelian Randomization and Cardiovascular Remodeling*, unites six pioneering studies that exemplify MR’s transformative potential in cardiovascular research. Here, we contextualize their contributions, identify unifying themes, and chart a roadmap for future inquiry.

## 2 The Power of MR in cardiovascular research

MR’s ability to mitigate confounding and reverse causality has positioned it at the forefront of causal inference. By integrating genetic, metabolomic, and clinical data, the studies in this Research Topic illuminate novel pathways in cardiovascular remodeling.

### 2.1 Gut-heart axis and metabolic mediators

Guan et al. (2025) employed MR mediation analysis to delineate a causal chain linking gut microbiota dysbiosis (*Prevotella copri* and *Alistipes putredinis*) to heart failure via the metabolite *Campesterol*. This work not only validates the gut microbiome’s role in lipid metabolism but also pioneers a framework for identifying metabolite-mediated therapeutic targets. Their findings underscore the importance of large-scale genomic datasets in overcoming limitations of traditional observational studies.

### 2.2 Senescence as a driver of cardiac dysfunction

Bian et al. (2024) merged single-cell RNA sequencing with MR to implicate *CDKN1A*—a senescence-related gene—in cardiomyocyte aging and heart failure progression. By identifying methylation sites (e.g. cg03714916) as modifiable risk factors, this study bridges epigenetics and clinical outcomes, offering a blueprint for targeting cellular senescence in age-related cardiovascular diseases.

### 2.3 Hematological traits and metabolic disease across ancestries

The MR analysis by Soremekun et al. (2025) revealed ancestry-specific associations between erythrocyte indices (e.g. mean corpuscular hemoglobin) and type 2 diabetes in African populations. These findings challenge conventional paradigms of the pathogenesis of type 2 diabetes and emphasize the need for trans-ancestry studies to address healthcare disparities.

### 2.4 Metabolomic signatures in vascular pathology

Guo et al. (2024) identified 29 metabolites and ratios, including uridine-pseudouridine and glycochenodeoxycholate sulfate, as causal factors in abdominal aortic aneurysm. Their work expands the metabolomic Frontier in vascular biology, highlighting bile acid signaling and nucleotide metabolism as critical regulators of vascular integrity.

### 2.5 Pharmacological and lifestyle interventions

Yang et al. (2025) demonstrated butylphthalide’s dual efficacy in reducing carotid plaque burden (via anti-inflammatory and MMP suppression) and improving neurological outcomes, while another MR analysis from this research group linked raw vegetable intake to reduced risk of atherosclerotic cardiovascular disease (Xu et al., 2024). These studies exemplify how MR can guide both drug development and public health strategies.

## 3 Emerging paradigms and unanswered questions

The collective findings of this Research Topic reveal three transformative themes.

### 3.1 From correlation to mechanism

MR’s strength lies in its ability to infer causality, yet mechanistic validation remains critical. For instance, *Campesterol*’s role in heart failure warrants exploration in preclinical models to clarify its impact on myocardial lipid metabolism. Similarly, *CDKN1A*’s regulatory network in senescence demands single-cell epigenomic profiling to identify downstream targets.

### 3.2 Ancestry-informed precision medicine

The divergent diabetes-hematology associations between African and European cohorts underscore the limitations of Eurocentric genomic databases. Future MR studies must prioritize diverse populations to uncover ancestry-specific pathways and optimize therapeutic strategies.

### 3.3 Multi-omics integration

While individual studies focused on genomics or metabolomics, integrating proteomics, microbiomics, and clinical data could resolve complex interactions. For example, combining gut microbiome profiles with cardiac proteomic datasets might elucidate how microbial metabolites modulate *CDKN1A*-driven senescence.

## 4 Future directions: bridging discovery to therapy

To translate MR-derived insights into clinical impact, we propose.

### 4.1 Preclinical platforms for causal validation

Organoid models and CRISPR-based screens could test hypotheses generated by MR (e.g. *Campesterol* inhibition in heart failure) while minimizing ethical and logistical challenges of human trials.

### 4.2 Trans-ancestry consortia

Large-scale collaborations, such as the Global Cardiovascular MR Initiative, should harmonize genomic and metabolomic data across ancestries to identify universal versus population-specific therapeutic targets.

### 4.3 Precision nutrition and digital health

MR can personalize dietary recommendations (e.g. uridine-rich diets for aortic aneurysm prevention) and integrate with digital tools (e.g. wearable biomarkers) to monitor intervention efficacy in real time.

## 5 Conclusion

This Research Topic exemplifies MR’s pivotal role in advancing cardiovascular medicine—from uncovering causal pathways to guiding targeted interventions. As the field evolves, interdisciplinary collaboration will be essential to harness multi-omics data, address health inequities, and transform causal insights into therapies that halt or reverse cardiovascular remodeling. The journey from genetic variant to bedside innovation has begun, and MR is leading the way.
